# PHQ-8 Days: a measurement option for DSM-5 Major Depressive Disorder (MDD) severity

**DOI:** 10.1186/1478-7954-9-11

**Published:** 2011-04-28

**Authors:** Satvinder S Dhingra, Kurt Kroenke, Matthew M Zack, Tara W Strine, Lina S Balluz

**Affiliations:** 1 Northrop Grumman Information Systems, CDC Programs, University Office Park/Harvard Building, 3375 Northwest Expressway, Atlanta, GA 30341, USA; 2 Center for Excellence in Implementing Evidence-Based Practice, Roudebush VA Medical Center, Indiana University and Regenstrief Institute, 1481 W 10th St, Indianapolis, IN 46202, USA; 3 Division of Adult and Community Health, National Center for Chronic Disease Prevention and Health Promotion, Centers for Disease Control and Prevention, 1600 Clifton Rd., NE, Mailstop E-K51, Atlanta, Georgia 30333, USA; 4 Division of Behavioral Surveillance, Public Health Surveillance Office, Office of Surveillance, Epidemiology, and Laboratory Services, Centers for Disease Control and Prevention, 1600 Clifton Rd., NE, Mailstop E-97, Atlanta, Georgia 30333, USA

## Abstract

**Background:**

Proposed draft diagnostic criteria for the fifth edition of the *Diagnostic and Statistical Manual of Mental Disorders *(DSM-5) suggest that dimensional assessments can supplement dichotomous diagnoses by incorporating measures of severity, frequency, and duration, providing the ability to monitor changes in symptoms over time and to guide appropriate treatment.

**Methods:**

This report is based on data from the Behavioral Risk Factor Surveillance System 2006 from 198,678 survey participants who responded to all eight Patient Health Questionnaire (PHQ-8) items. We evaluated use of the days version of the PHQ-8 to determine an optimal cut-point for identifying respondents with depression and to evaluate the performance characteristics of the PHQ-8 at this cut-point.

**Results:**

A PHQ-8 score of 55 or more days was determined to be the optimal cut-point when compared to the DSM-derived PHQ-8 algorithm for a major depressive episode (five or more symptoms present "more than half the days," at least one of which must be anhedonia or depression). In the full sample, the sensitivity and the specificity of this cut-point were 0.91 (0.90-0.93) and 0.99 (0.99-0.99), respectively.

**Conclusion:**

The days version of the PHQ-8 may be a valuable dimensional alternative to the traditional PHQ-8 by offering finer granularity of dimensionality (a score of 0 to 112).

## Background

*Diagnostic and Statistical Manual of Mental Disorders *(DSM) criteria are presently designed to establish categorical diagnoses, distinguishing those with a particular mental disorder from those without such a disorder [1]. DSM criteria are currently less useful for measuring psychiatric symptoms and disorders on a continuum. Major depressive disorder is classified as a mood disorder, with diagnosis hinging on the presence of a single episode or of recurrent major depressive episodes (MDE) [1]. The gold standard for a diagnosis of depression is the Structural Clinical Interview (SCID), a diagnostic interview based on DSM criteria that requires clinical expertise to administer. It yields a dichotomous outcome, the presence or absence of MDE, for the past month (current), past year, or over a lifetime, based on the presence of five or more of the nine DSM criteria, provided that anhedonia or depression was present [1,2].

The proposed draft diagnostic criteria for the fifth edition of the DSM (DSM-5; http://www.dsm5.org) suggest that graded, dimensional assessments can supplement dichotomous diagnoses. Furthermore, dimensional assessments incorporating measures of severity, frequency, and duration may help psychiatric research, epidemiology, and clinical services to not only better monitor changes in respondents' symptoms over time but also to guide the choice of appropriate population and clinical interventions [3]. Categorical and dimensional approaches are fundamentally equivalent with no one right approach. Advocates of both approaches may well be right, but in different circumstances [4,5].

Given the time and expense required to administer the SCID, epidemiological studies instead use either structured interviews designed for trained lay interviewers (e.g., the Composite International Diagnostic Interview [CIDI], the Diagnostic Interview Schedule [DIS]) or self-report questionnaires [6-8]. Such self-report questionnaires (e.g., the Center for Epidemiologic Studies of Depression Scale [CES-D], versions of the Patient Health Questionnaire [PHQ-9, PHQ-8], the Beck Depression Inventory [BDI], and other measures) [9-11] measure symptoms and mood to provide evidence for a disorder (defined as "something wrong with a patient that is of clinical significance") rather than for a diagnosis (defined as "an expert opinion that a disorder is present") [4]. Nonetheless, self-report questionnaires such as the 9-item (PHQ-9) and the 8-item (PHQ-8) Patient Health Questionnaire depression measure can provide a dimensional assessment for depression because they are scored by summing how often a number of typical depressive symptoms occur [5,9]. A PHQ-8 score of ≥10 can also yield a categorical diagnosis of clinically significant depression and is more convenient to use than a DSM-IV diagnostic algorithm [9].

A recent revision to the PHQ-8 (referred to as the PHQ-8 Days) used in the Behavioral Risk Factor Surveillance System (BRFSS) survey adds further dimensionality to the PHQ-8 by asking the number of days in the past 14 days the respondent experienced each of the eight depressive symptoms, yielding 0 to 112 total days [12]. In this study, we determine the optimal cut-point of the PHQ-8 Days scale for identifying respondents experiencing major depression during the past two weeks, and then evaluate the performance characteristics of the PHQ-8 Days at this cut-point. We estimate the robustness of its receiver operating characteristic (ROC) curve and compare the prevalence of major depression at this cut-point (positive test frequency) with that based on the proportion of PHQ-8 respondents meeting the DSM algorithm criteria for MDE. We also demonstrate the fine granularity of the PHQ-8 Days scale by lifetime diagnosis of anxiety and depression and multiple domains of health-related quality of life. Assessment of the PHQ-8 Days scale in this large epidemiological study may provide further evidence of its utility as a dimensional measure of depression in population-based research.

## Methods

### Behavioral Risk Factor Surveillance System survey (BRFSS)

We analyzed data from the 2006 BRFSS survey. The BRFSS is a population-based, state surveillance system using ongoing, random-digit-dialed telephone surveys of noninstitutionalized US residents aged 18 years or older that monitors the prevalence of key health- and safety-related behaviors and characteristics [13,14]. During the 2006 survey, trained interviewers in 41 states and territories administered the Anxiety and Depression Module, which includes the PHQ-8 [12]. Weighting of BRFSS data is designed to make the total number of cases equal to the number of people in the state who are age 18 and older. In the BRFSS, such post-stratification serves as an adjustment for noncoverage and nonresponse and forces the total number of cases to equal population estimates for each geographic region, usually a state for the BRFSS. The median response rate among all states and territories, based on Council of American Survey and Research Organizations (CASRO) guidelines, was 51.4% (range: 35.1%-66.0%) in 2006, 50.6% (range: 26.9%-65.4%) in 2007, and 53.3% (range: 35.8%-65.9%) in 2008. The median cooperation rate was 74.5% (range: 56.9%-83.5%) in 2006, 72.1% (range: 49.6%-84.6%) in 2007, and 75.0% (range: 59.3%-87.8%) in 2008. Surveillance methodology, design, implementation, and response rates are available at: http://www.cdc.gov/brfss/technical_infodata/2002QualityReport and http://www.cdc.gov/BRFSS/technical_infodata/index.htm.

There were 198,678 respondents from the 38 states, Washington, DC, Puerto Rico, and the US Virgin Islands who completed all of the PHQ-8.

## Measures

### Patient Health Questionnaire eight-item depression scale (PHQ-8)

The PHQ-8 response set was standardized to make it similar to other BRFSS questions by asking the number of days in the past two weeks the respondent had experienced each of the eight out of nine DSM criteria symptoms. In previous BRFSS analyses, the modified response set had been converted back to the original response set: 0 to 1 day = 'not at all,' 2 to 6 days = 'several days,' 7 to 11 days = 'more than half the days,' and 12 to 14 days = 'nearly every day,' with points (0 to 3) assigned to each category, respectively [12]. The scores for each item are summed to produce a total score between 0 and 24 points. A total score of 0 to 4 represents no significant depressive symptoms; 5 to 9, mild symptoms; 10 to 14, moderate symptoms; 15 to 19, moderately severe symptoms; and 20 to 24, severe symptoms [9]. *Current depression *is defined in two ways: 1) a PHQ-8 DSM-derived algorithm diagnosis of *major depression *(≥ five symptoms present 'more than half the days,' with at least one symptom being anhedonia or depression) or *other depression *(two to four symptoms, including depressed mood or anhedonia, are required to be present 'more than half the days'); 2) a PHQ-8 score of ≥10, which has an 88% sensitivity and 88% specificity for major depression, and, regardless of diagnostic status, typically represents clinically significant depression [9].

### Lifetime diagnosis of anxiety or depressive disorders

Two questions were asked about lifetime diagnosis: "Has a doctor or other health care provider ever told you that you have an anxiety disorder (including acute stress disorder, anxiety, generalized anxiety disorder, obsessive-compulsive disorder, panic attacks, panic disorder, phobia, post-traumatic stress disorder, or social anxiety disorder)?" and "Has a doctor or other health care provider ever told you that you have a depressive disorder (including depression, major depression, dysthymia, or minor depression)?"

### Health-related quality of life and other items

Three health-related quality of life (HRQoL) questions with demonstrated validity and reliability for population health surveillance were examined [15-17]. The three questions involved respondents' self-assessment of their health over the previous 30 days:

1) Physical health: "How many days was your physical health, which includes physical illness or injury, not good?"

2) Mental health: "How many days was your mental health, which includes stress, depression, and problems with emotions, not good?"

3) Activity limitations: "Are you limited in any way in any activities because of physical, mental, or emotional problems?"

### Sociodemographic characteristics

Sociodemographic information was obtained for each respondent. We assessed the extent to which seven sociodemographic characteristics (sex, age, race, education, employment status, annual household income, and marital status) were associated with major depression as determined by participants' responses to the PHQ-8.

## Analysis

In this study, the condition of interest is MDE as defined by DSM-IV-derived PHQ-8 algorithm "gold standard" criteria (five or more depressive symptoms present 'more than half the days,' and at least one of which must be anhedonia or depression). Sensitivity is the proportion of persons with this condition ascertained by a test to have that condition. Specificity is the proportion of persons without this condition ascertained by a test not to have the condition. Youden's J index (YJI), one measure of combined test validity, is the sum of the sensitivity and the specificity minus 1. The test is the PHQ-8 Days cut-point with the highest simultaneous values for sensitivity and specificity (maximum YJI) among respondents indicating ≥ seven days of either anhedonia or depression (n = 22,542).

We ascertained this cut-point by converting all BRFSS weights to integer weights to obtain the ROC curve and area under the curve (AUC) over the range of test values (0 to 112 days). The ROC curve summarizes test validity measures over the range of the test values and plots the sensitivity of the test on the vertical axis versus (1 - specificity) on the horizontal axis. The AUC is a measure of accuracy of a test instrument, with AUCs between 0.5 and 0.7 considered as reflecting low accuracy; between 0.7 and 0.9, moderate accuracy; and those above 0.9, high accuracy [15].

We assessed the prevalence of major depression by (a) using this PHQ-8 Days cut-point and (b) according to the DSM-derived PHQ-8 algorithm criteria for MDE by sex, age, race, education, employment status, annual income, and marital status. We also estimated the mean number of PHQ-8 Days by three measures of HRQoL and lifetime diagnosis of anxiety and depression. We used SPSS, Version 17.0 (SPSS Inc., Chicago, IL) with the complex sampling module for all analyses.

## Results

**Figure 1:**
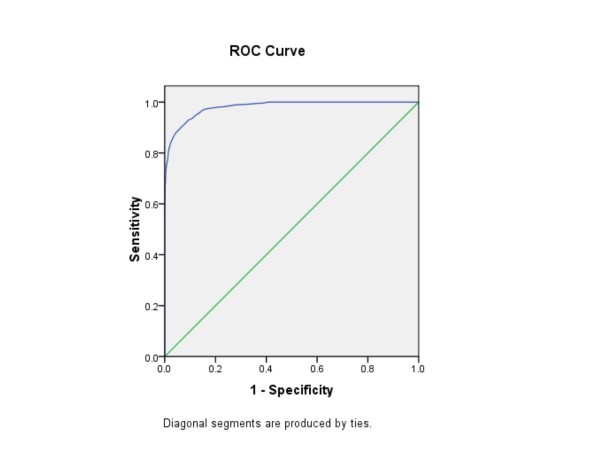
**Receiver Operating Characteristic (ROC) curve plotted for Sensitivity and 1 - Specificity for each number of PHQ-8 Days (0 to 112 days) in identifying respondents indicating ≥ 7 days of either anhedonia or depression (n = 22,542), with major depressive episode (MDE) as defined by a DSM-IV-derived PHQ-8 criteria of ≥ 5 symptoms present 'more than half the days' and at least one of which must be anhedonia or depression**.

A PHQ-8 score of 55 or more days was determined to be the optimal cut-point when compared to the DSM-derived PHQ-8 MDE algorithm (≥ five symptoms present 'more than half the days' and at least one of which must be anhedonia or depression). While the Youden's J Index was similar (0.836) over a narrow range of cut-points from 53 to 56, a cut-point of 55 or higher was selected (Table 1). AUC for the range of the test values (0-112) was 0.98 (Figure 1). Among the full sample of 198,678 people who responded to all eight PHQ-8 questions, the sensitivity and the specificity of a PHQ-8 Days cut-point of 55 or higher were 0.91 (0.90-0.93) and 0.99 (0.99-0.99), respectively.

**Table 1:** Operating characteristics of the number of PHQ-8 Days (range: 0 to 112 days) for identifying a major depressive episode as defined by DSM-IV-derived PHQ-8 criteria^†^

PHQ-8 Days	Sensitivity	Specificity	Youden's J Index
<50	≥0.969	≤0.849	0.818
≥50	0.955	0.868	0.823
≥51	0.945	0.882	0.827
≥52	0.936	0.892	0.828
≥53	0.929	0.907	0.836
≥54	0.923	0.913	0.836
≥55	0.913	0.922	0.835
≥56	0.904	0.932	0.836
≥57	0.882	0.953	0.835
≥58	0.874	0.960	0.834
≥59	0.863	0.966	0.829
≥60	≤0.850	≥0.972	0.822

^†^DSM-IV-derived PHQ-8 criterion: ≥ 5 symptoms present 'more than half the days' and at least one of which must be anhedonia or depression. Operating characteristics determined in respondents indicating ≥ 7 days of either anhedonia or depression (n = 22,542).

The prevalence estimates of MDE, based on the PHQ-8 Days optimal cut-point of 55 or more days, did not differ statistically significantly from the prevalence estimates of DSM-derived PHQ-8 MDE by sex, age, race, education, employment status, annual income, and marital status (Table 2).

**Table 2:** Prevalence of current major depressive episode according to DSM-IV-derived PHQ-8 criterion^† ^and the alternative PHQ-Days algorithm^‡ ^by sociodemographic characteristics (unweighted numbers, weighted proportions per 100)

Sociodemographic characteristics	Unweighted number	^†^Major depression DSM criterion prevalence	95% Confidence Interval of prevalence	^‡^Major depression PHQ-Days algorithm prevalence	95% Confidence Interval of prevalence
**Unweighted n**	198,678	8,476		8,970	
**Overall**		4.2	4.0 - 4.4	4.4	4.2 - 4.6
**Sex**					
Female	122,390	4.9	4.7 - 5.2	5.2	4.9 - 5.5
Male	76,288	3.4	3.1 - 3.7	3.5	3.2 - 3.8
**Age**					
18 - 24	9,186	3.7	3.1 - 3.4	4.0	3.3 - 4.7
25 - 34	24,493	4.3	3.8 - 4.9	4.6	4.0 - 5.2
35 - 44	34,910	4.7	4.2 - 5.2	4.8	4.3 - 5.3
45 - 54	42,321	5.2	4.8 - 5.7	5.4	5.0 - 5.9
55 - 64	39,235	4.7	4.2 - 5.2	4.9	4.4 - 5.4
≥65	47,161	2.1	1.8 - 2.4	2.3	2.1 - 2.6
**Race/ethnicity**					
White	153,642	3.9	3.7 - 4.1	4.0	3.8 - 4.3
Black	15,819	4.8	4.2 - 5.4	5.2	4.6 - 5.8
Hispanic	15,602	4.7	4.0 - 5.5	4.9	4.2 - 5.7
Other	11,955	5.3	4.5 - 6.4	5.6	4.7 - 6.7
**Education**					
<High school	18,776	8.1	7.2 - 9.1	8.7	7.8 - 9.6
High school	58,191	5.0	4.6 - 5.3	5.2	4.9 - 5.6
>High school	121,441	3.0	2.8 - 3.2	3.2	2.9 - 3.4
**Employment status**					
Employed for wages	96,650	2.4	2.2 - 2.7	2.6	2.4 - 2.9
Self-employed	18,924	2.7	2.2 - 3.4	2.8	2.3 - 3.5
Out of work >1 year	3,242	13.8	11.6 - 16.3	14.2	12.1 - 16.7
Out of work <1 year	4,106	9.6	8.0 - 11.5	10.2	8.6 - 12.0
Homemaker	15,848	3.9	3.3 - 4.6	3.9	3.3 - 4.6
Student	4,053	3.8	2.7 - 5.2	3.7	2.7 - 5.2
Retired	44,496	2.1	1.9 - 2.4	2.3	2.1 - 2.6
Unable to work	11,006	26.8	24.9 - 28.7	28.4	26.5 - 30.3
**Marital status**					
Married	112,779	2.9	2.7 - 3.1	3.0	2.8 - 3.2
Divorced	28,362	8.3	7.7 - 9.1	8.7	8.0 - 9.5
Widowed	22,173	5.1	4.4 - 6.0	5.4	4.7 - 6.3
Separated	4,440	12.0	10.1 - 14.2	12.7	10.8 - 15.0
Never married	25,046	4.8	4.3 - 5.4	5.1	4.6 - 5.7
Member of unmarried couple	5,236	5.7	4.5 - 7.1	6.3	5.0 - 7.9

^†^DSM-IV-derived PHQ-8 criterion: ≥ 5 symptoms present 'more than half the days' and at least one of which must be anhedonia or depression.

^‡^Major depression PHQ-8 Days algorithm: 55 or more PHQ-8 Days.

Respondents with disabilities reported more than twice as many PHQ-8 Days, 25.8 (25.2-26.3), as respondents without disability, 10.3 (10.1-10.5; Table 3). As the number of mentally unhealthy days (Table 4) and physically unhealthy days (Table 5) increased, mean PHQ-8 Days and the weighted prevalence of major depression also increased.

**Table 3:** Mean number of PHQ-8 Days by disability ("Are you limited in any way in any activities because of physical, mental, or emotional problems?")

Disability status	Unweighted (n)	Weighted proportion (%)	95% Confidence Interval	Mean PHQ-8 Days	95% Confidence Interval
No	151,546	80.1	79.7 - 80.5	10.3	10.1 - 10.5
Yes	46,559	19.9	19.5 - 20.3	25.8	25.2 - 26.3

**Table 4:** Unweighted counts, weighted proportions, and mean PHQ-8 days by mentally unhealthy days from BRFSS 2006

Number of mentally unhealthy days	Un-weighted (n)	Weighted proportion (%)	95% Confidence interval	Mean PHQ-8 Days	95% Confidence Interval	Weighted prevalence of major depression (%)	95% Confidence Interval
0	133,812	65.8	65.3 - 66.2	7.7	7.6 - 7.9	0.6	0.5 - 0.7
1 - 6	38,119	19.0	18.6 - 19.4	13.9	13.5 - 14.3	1.8	1.5 - 2.2
7 - 10	9,279	4.7	4.5 - 4.9	24.2	23.0 - 25.3	8.3	7.1 - 9.6
11 - 15	6,804	3.3	3.1 - 3.5	33.4	31.9 - 34.9	16.7	14.6 - 19.1
16 - 20	2,870	1.4	1.3 - 1.6	41.2	39.3 - 43.2	25.0	21.8 - 28.5
21 - 30	13,609	5.8	5.6 - 6.0	49.2	47.9 - 50.5	37.3	35.4 - 39.3

**Table 5:** Unweighted counts, weighted proportions, and mean PHQ-8 days by physically unhealthy days from BRFSS 2006.

Number of physically unhealthy days	Un-weighted (n)	Weighted proportion (%)	95% confidence interval	Mean PHQ-8 Days	95% confidence interval	Weighted prevalence of major depression (%)	95% confidence interval
0	141,421	64.4	63.9 - 64.9	9.4	9.3 - 9.6	1.9	1.7 - 2.1
1 - 6	41,479	20.4	20.1 - 20.8	14.6	14.1 - 15.0	3.6	3.2 - 4.1
7 - 10	9,553	4.1	3.9 - 4.2	21.1	20.1 - 22.0	8.0	6.9 - 9.3
11 - 15	7,059	3.0	2.8 - 3.1	27.6	26.0 - 29.2	12.8	11.0 - 15.0
16 - 20	2,658	1.1	1.0 - 1.2	32.0	29.7 - 34.2	14.8	12.3 - 17.7
21 - 30	19,664	7.0	6.8 - 7.2	34.1	32.8 - 35.3	20.1	18.7 - 21.6

Mean PHQ-8 Days increased markedly with changes in current depression status (none, other, and major) and in lifetime depression status (No to Yes) but not with changes in lifetime anxiety status (No to Yes) except in those without current depression (Table 6).

**Table 6:** Unweighted counts, weighted mean PHQ-8 days, and prevalence estimates by eight mutually exclusive categories of depression severity (major, other, and none) and lifetime depression and/or anxiety from BRFSS 2006

	Lifetime diagnosis						
						
Current depression status	Un-weighted (n)	Weighted proportion (%)	95% confidence interval	Mean PHQ-8 Days	95% confidence interval	Depression status	Anxiety status
None	No	No	146,048	76.1	75.7 - 76.5	7.6	7.4 - 7.7
None	No	Yes	7,372	3.5	3.3 - 3.7	13.7	13.0 - 14.4
None	Yes	No	16,004	6.7	6.5 - 7.0	15.6	15.1 - 16.1
None	Yes	Yes	10,296	4.6	4.4 - 4.7	20.8	20.1 - 21.4
Other	No	No	5,491	3.1	2.9 - 3.3	36.4	35.8 - 37.0
Other	No	Yes	585	0.3	0.2 - 0.3	38.2	36.1 - 40.3
Other	Yes	No	1,674	0.8	0.7 - 0.9	42.3	41.2 - 43.4
Other	Yes	Yes	1,733	0.8	0.7 - 0.9	44.4	43.2 - 45.5
Major	No	No	2,297	1.3	1.2 - 1.4	73.9	72.3 - 75.6
Major	No	Yes	380	0.2	0.1 - 0.2	74.0	71.2 - 76.8
Major	Yes	No	1,808	0.8	0.7 - 0.9	77.4	75.7 - 79.2
Major	Yes	Yes	3,853	1.8	1.7 - 1.9	80.8	79.5 - 82.1

## Discussion

To the best of our knowledge, this is the first study to examine and extend the proposed DSM-5 dimensionality available using PHQ-8 in its current response format. A PHQ-8 Days cut-point of 55 or more days (provided anhedonia or depression was present seven or more days) best identified respondents with MDE derived from a DSM-based PHQ-8 algorithm. AUC for the range of test values reflected high accuracy [18]. Prevalence estimates of MDE based on the cut-point of 55 or more days were not statistically significantly different from those derived from the DSM-based PHQ-8 algorithm when stratified by seven sociodemographic characteristics. Prevalence estimates of MDE, based on the cut-point of 55 or more days for all categories of sociodemographic characteristics examined, were higher than those of MDE derived from the DSM-based PHQ-8 algorithm, except for no difference in prevalence among homemakers. Prevalence estimates at 56 or more days would have been closer to the DSM-derived MDE algorithm estimates. It is noteworthy that beyond two weeks, MDE prevalence estimates from the BRFSS of 4.4% and 4.2%, based on the cut-point of 55 days or more and from the DSM-based PHQ-8 algorithm, respectively, are in close proximity to prevalence rates of past 30-day major depression found in other studies and in a systematic review of the literature [19-21].

Besides the robust operating characteristics of PHQ-8 Days at the cut-point of 55 or more days, this study had other key findings. First, even among respondents without current depression, mean PHQ-8 Days increased significantly in a stepwise manner from 7.6 days (7.4-7.7) for respondents without lifetime depression or anxiety to 20.8 days (20.1-21.4) for respondents with lifetime depression and anxiety. Among respondents with current other or current major depression, a lifetime history of depression but not anxiety increased mean PHQ-8 days statistically significantly. Second, the dimensional scale of PHQ-8 days increased with both physically and mentally unhealthy days, especially with the latter. Mean PHQ-8 Days increased from about nine days at a level of 0 physically unhealthy days to 28 days at 11-15 physically unhealthy days and to 34 days at 21-30 physically unhealthy days. Mean PHQ-8 days increased from about 8 days at 0 mentally unhealthy days to 33 days at 11-15 mentally unhealthy days and to 44 days at 21-30 mentally unhealthy days.

The PHQ-8 Days version may be a valuable dimensional alternative to the traditional PHQ in several respects for psychiatric epidemiology and clinical services. First, its finer granularity (scores from 0 to 112 for PHQ-8 vs. 0 to 24; or 0 to 126 for PHQ-9 vs. 0 to 27) may increase sensitivity to change when monitoring depression longitudinally in clinical trials or cohort studies. Second, a quantitative response format ("number of days") and a standardized recall period provide greater uniformity and ease of translation, whereas the current verbal response options such as "several," "more than half," or "nearly every day" may be more susceptible to variable interpretations when translated into different languages or used across multiple cultures. Third, the current verbal response set using these less well-defined words and phrases is only an ordinal level, not an interval level, of measurement. Fourth, entering the number of days is easier to use in automated data gathering such as interactive voice recorded (IVR) calls. A potential disadvantage of using "number of days" is that some respondents may not be able to provide a specific number of days so that an interviewer may have to interpret what the respondent reply means or to interpolate when the respondent provides a range rather than a discrete number.

Some of these as well as other factors make the PHQ-8 Days version a useful option for public health research and surveillance. Individuals assessed in the general population typically have fewer and less severe depressive symptoms than patients evaluated in clinical settings; in this case, the greater range of the PHQ-8 Days version might make it more sensitive to subthreshold symptoms, with fewer concerns about a floor effect. Besides being valuable for capturing the full spectrum of depressive symptoms in the general population, this feature may also be useful in detecting low levels of symptoms that may occur in the wake of man-made or natural disasters and in monitoring mental health following such traumatic events. The reduction in cultural and language variability with the quantitative response set and the greater ease-of-use with automated data gathering may be particularly useful for large population-based surveys.

Key strengths of this study are that the survey populations in participating states were reasonably representative of the state populations and that sample sizes were large enough to analyze positive test frequency in seven sociodemographic subgroups. Although the PHQ-8 was used in the BRFSS, the concept of PHQ days can likely be applied to the PHQ-9 as well. The cut-points on the PHQ-8 and PHQ-9 are identical, and either is a valid measure of depression severity [22,23]. The PHQ-8 omits the ninth item of the PHQ-9 (which asks about thoughts of death or self-harm) and is often used in epidemiological studies where professional follow-up is unavailable or impractical, and in clinical research studies where depression is a secondary rather than primary outcome. Almost all of the positive responses to this ninth item represent passive thoughts of death rather than suicidal ideation.

Studies based on BRFSS data in general and the depression and anxiety module in particular have some inherent limitations. First, they are representative of only households with landline telephones included in BRFSS surveys. If respondents in currently excluded households without telephones or with only cell phones answer the PHQ-8 Days questions differently from respondents in households with landline telephones, the prevalence estimates of MDE may be biased compared to that from interviews in all households, but this difference should not affect the test validity of the PHQ-8 Days measure proposed here. Second, because BRFSS data are based on subjective responses of survey participants, recall bias and biases related to the perceived social desirability of certain responses may affect their accuracy. Third, BRFSS and the "gold standard" DSM diagnostic algorithm for MDE to which the PHQ-8 Days version is compared are unable to address both the inclusion criteria for symptoms that cause clinically significant distress or impairment in social, occupational, or other important areas of functioning and the exclusion criteria for episodes due to the direct physiological effects of a substance or antidepressant intervention (e.g., a drug of abuse, a medication, or other treatment). Furthermore, the BRFSS and this "gold standard" do not account for those being successfully treated and asymptomatic at the time of the survey. Despite these limitations, other BRFSS estimates have been shown to be valid and reliable when compared with estimates derived from national household survey data [24,25]. BRFSS surveys are a cost-effective and timely means of collecting state and local data, and BRFSS data are often the only data source with which states and communities can assess local health conditions and track progress toward improving those conditions.

We have demonstrated the ease of using the PHQ-8 Days responses not only to create a highly granular dimensional measure but also to identify a categorical cut-point for major depressive episode. Additional cut-points for other categories of depression severity could easily be identified, giving the psychiatric epidemiological and services community the much-needed granularity and flexibility to detect changes and help monitor changes in respondents' symptoms over time as well as providing additional data to help guide the choice of appropriate interventions [3].

### Disclaimer

The findings and conclusions in this article are those of the authors and do not necessarily represent the official position of the Centers for Disease Control and Prevention.

## Competing interests

The authors declare that they have no competing interests.

## Authors' contributions

SSD designed the study and performed statistical analyses. SSD, KK, MMZ, TWS, and LSB drafted the manuscript and approved the final version. SSD accepts full responsibility for the work and the conduct of the study, had access to the data, and controlled the decision to publish. All authors read and approved the manuscript.
